# X-Linked Hypophosphatemia in a Family Cohort: Clinical Variability, Genetic Confirmation and Modern Therapeutic Perspectives

**DOI:** 10.3390/jcm14217496

**Published:** 2025-10-23

**Authors:** Oana Popa, Melania Balaș, Ioana Golu, Daniela Amzăr, Carmen Dorogi, Mihaela Vlad

**Affiliations:** 1 Department of Internal Medicine II, University Clinic of Endocrinology, Centre for Molecular Research in Nephrology and Vascular Disease, Faculty of Medicine, “Victor Babeș” University of Medicine and Pharmacy Timișoara, 300041 Timișoara, Romania; oana.taban@umft.ro (O.P.); balas.melania@umft.ro (M.B.); golu.ioana@umft.ro (I.G.); amzar.daniela@umft.ro (D.A.); vlad.mihaela@umft.ro (M.V.); 2 Department of Endocrinology, “Pius Brânzeu” County Emergency Clinic Hospital Timișoara, 300723 Timișoara, Romania

**Keywords:** X-linked hypophosphatemia, PHEX mutation, burosumab, FGF23, case series, multidisciplinary care

## Abstract

**Background/Objectives:** X-linked hypophosphatemia (XLH) is the most common form of inherited rickets, caused by pathogenic mutations in the PHEX gene (phosphate-regulating endopeptidase homolog, X-linked). These mutations increase fibroblast growth factor 23 (FGF23) activity, resulting in renal phosphate wasting and defective bone mineralization. The disorder manifests with variable skeletal, dental, and extraskeletal involvement. Conventional therapy with oral phosphate and active vitamin D offers limited benefit, whereas burosumab, an anti-FGF23 monoclonal antibody, has transformed disease management. **Methods:** The index case, a 43-year-old woman, remained undiagnosed until adulthood, leading to severe deformities, osteoarthritis, chronic pain, and complete edentulism. Her 55-year-old sister presented with a milder phenotype. The 20-year-old nephew, diagnosed in childhood and intermittently treated with phosphate and alfacalcidol, developed short stature, genu varum, and early degenerative joint disease. Following genetic confirmation, he began burosumab therapy, which normalized phosphate metabolism, reduced pain, and improved mobility. **Results:** XLH demonstrates marked intrafamilial phenotypic variability despite identical PHEX mutations. In this series, delayed recognition in adults led to irreversible skeletal deformities, osteoarthritis, and dental loss, whereas earlier diagnosis in the younger patient allowed timely intervention. Conventional therapy only partially mitigated complications, while burosumab achieved rapid biochemical correction and symptomatic improvement. This contrast highlights the importance of early genetic testing, family screening, and prompt initiation of targeted treatment. **Conclusions:** This family cluster underscores the critical need for early diagnosis, genetic confirmation, cascade screening, and lifelong multidisciplinary care. Burosumab represents a therapeutic paradigm shift in XLH, capable of altering disease trajectory when initiated early.

## 1. Introduction

X-linked hypophosphatemia (XLH) is the most common inherited form of rickets, with an estimated incidence of 1 in 20,000–70,000 live births. It results from pathogenic variants in the PHEX gene [1,2], which lead to excessive fibroblast growth factor 23 (FGF23) activity, renal phosphate wasting, and impaired bone mineralization [3].

XLH manifests in both children and adults with heterogeneous skeletal, dental, and extraskeletal involvement, typically resulting in chronic morbidity and a sustained impact on quality of life [4].

The increasing availability of long-term registry data has revealed the clinical heterogeneity of XLH and offered valuable insight into its natural history, helping to guide best practices in patient care. These registries underscore the importance of early diagnosis, timely initiation of therapy, and coordinated multidisciplinary management involving endocrinology, nephrology, orthopedics, dentistry, audiology, and psychosocial support [5].

The latest international, evidence-based clinical practice guidelines provide a comprehensive framework for the diagnosis and management of XLH across the lifespan. In children, XLH should be suspected in cases of rickets, poor growth velocity, dental abnormalities, or craniosynostosis—particularly when hypophosphatemia and renal phosphate wasting are present without calcium or vitamin D deficiency. In adolescents and adults with closed growth plates, findings such as osteomalacia, pseudofractures, early-onset osteoarthritis, enthesopathies, or recurrent dental abscesses in the setting of hypophosphatemia should raise clinical suspicion. Recommended diagnostic investigations include a thorough clinical evaluation, radiographic assessment of rickets or osteomalacic changes, and a comprehensive biochemical work-up. Laboratory testing should include fasting (in adults) or random (in children) serum phosphate, calcium, alkaline phosphatase, parathyroid hormone, and vitamin D metabolites (25-hydroxyvitamin D and, when available, 1,25-dihydroxyvitamin D). Measurement of intact FGF23 and urinary phosphate and calcium levels, with calculation of the tubular maximum for phosphate reabsorption (TmP/GFR), are also advised. Whenever possible, genetic confirmation of PHEX variants is strongly recommended [1].

Historically, treatment relied on conventional regimens consisting of multiple daily doses of oral phosphate salts combined with active vitamin D analogues. While this approach could partially control rickets and osteomalacia, it was limited by gastrointestinal intolerance and long-term complications such as secondary hyperparathyroidism and nephrocalcinosis [6].

A major therapeutic shift has occurred with the introduction of burosumab, a monoclonal antibody directed against FGF23, which directly targets the underlying pathophysiological mechanism. Both clinical trial evidence and real-world data have demonstrated superior biochemical and radiological responses, together with significant improvements in patient-reported quality of life, compared with conventional treatment [7] with phosphate and active vitamin D supplementation—of limited efficacy and burdened with substantial risk.

In X-linked hypophosphatemia, vitamin D plays a supportive but secondary role in the correction of mineral metabolism. As outlined by Rowe et al., excess fibroblast growth factor 23 (FGF23) suppresses renal 1α-hydroxylase activity, thereby reducing the conversion of 25-hydroxyvitamin D to its active 1,25-dihydroxy form, which in turn contributes to defective intestinal phosphate absorption and impaired bone mineralization. Consequently, even with adequate nutritional vitamin D levels, the disorder manifests as a form of vitamin D–resistant rickets [2]. According to Haffner et al., conventional therapy with oral phosphate and active vitamin D analogues (calcitriol or alfacalcidol) remains essential to enhance phosphate absorption and suppress secondary hyperparathyroidism, yet it does not address the primary FGF23-driven mechanism.

Burosumab is now endorsed as first-line therapy in both children and adults, given its demonstrated capacity to normalize phosphate homeostasis, heal rickets and osteomalacia, improve growth and enhance quality of life.

The new drug is recommended for children with XLH showing radiographic evidence of rickets and for symptomatic adolescents and adults with confirmed disease. It is administered by subcutaneous injection every two weeks in children and every four weeks in adults, with dosing adjusted to body weight. Follow-up includes monitoring of fasting serum phosphate, TmP/GFR, alkaline phosphatase, and clinical parameters such as growth, skeletal deformities, pain, and functional outcomes

Management principles emphasize lifelong, multidisciplinary follow-up coordinated by a metabolic bone disease specialist, with input from endocrinology, nephrology, orthopedics, dentistry, and, when appropriate, neurosurgery and audiology. Additional recommendations highlight the importance of structured monitoring of growth, skeletal deformities, and dental health; prevention and surveillance of hyperparathyroidism; individualized orthopedic management of deformities and enthesopathies; and careful supervision of affected women during pregnancy and lactation. Collectively, these guidelines reaffirm that early diagnosis, personalized therapy, and coordinated multidisciplinary care remain the cornerstones of favorable long-term outcomes in XLH [1].

In this article, we describe a family cluster comprising three related cases across two generations—S.G., a 43-year-old female index patient, her 20-year-old nephew, A.R. and his mother, A.D.—all harboring the same pathogenic *PHEX* exon 16 deletion. By contrasting their distinct clinical trajectories—ranging from long-standing, untreated disease in adulthood to partial responses under conventional therapy and the more recent initiation of targeted anti-FGF23 treatment—we aim to illustrate the phenotypic variability of XLH, highlighting the consequences of delayed diagnosis, and underscore the transformative impact of modern therapeutic strategies.

## 2. Materials and Methods

All cases were investigated and diagnosed in the Department of Endocrinology of the County Hospital Timișoara, Romania. This case series was conducted as a retrospective chart review and prospective follow-up of three related individuals diagnosed with X-linked hypophosphatemia at the Department of Endocrinology, County Hospital Timișoara, Romania. Patient selection was based on confirmed biochemical evidence of renal phosphate wasting, compatible clinical and radiological features of XLH, and familial aggregation suggesting X-linked inheritance. Following identification of the index case (S.G.), cascade testing was performed for first-degree relatives. All family members included in this report were confirmed to carry the same *PHEX* exon 16 deletion through genetic testing. Data collection involved comprehensive review of medical records, laboratory reports, imaging findings, and clinical documentation from hospital archives, supplemented by direct patient interviews and follow-up visits between 2022 and 2024. Clinical data were extracted from medical records, imaging reports, and laboratory results, and were verified by direct patient interviews during follow-up visits. Laboratory values were standardized against institutional reference ranges, and genetic testing CentoXome Solo was performed using next-generation sequencing with confirmatory multiplex ligation-dependent probe amplification (MLPA) at the Centogene Center in Rostock, Germany.

Laboratory assays were performed in the Laboratory Department of our hospital. The methodology for immunological analysis was enhanced chemiluminescence immunoassay method, chemiluminescence microparticle immunoassay and electroluminescence (Advia Centaur XPT, Siemens, Germany and Atellica IM 1600, Siemens, Munich, Germany). Biochemical testing was performed on a Vitros 7600 analyzer (MYCO Instrumentation Inc., Bonney Lake, WA, USA). Anterior neck ultrasounds were performed using Esaote Mylab Seven ultrasound machine (Esaote Mylab Seven, Software Version: 7.04.01, Genoa, Italy, 2017) with a 15 MHz linear probe. Thyroid nodules were graded according to the ACR TIRADS classification. Osteodensitometry (Eurotec DVision, Hagen, Germany, 2024) and radiological investigations were carried out in our hospital on the Radiology Department. Multidisciplinary evaluation was performed by teams including endocrinology, nephrology, orthopedics, rheumatology, and dentistry, ensuring comprehensive assessment of skeletal, renal, and dental outcomes. All patients signed an informed consent form that was approved by the Timișoara County Hospital ethics committee. This study was carried out in accordance with the Declaration of Helsinki’s Ethical Principles for Medical Research, agreeing to the use of their data that were collected during admission.

## 3. Case Series

### 3.1. Case 1—S.G., 43-Year-Old Female (Index Patient)

#### 3.1.1. Case Presentation

S.G., a 43-year-old woman, was the first member of the extended family to be formally diagnosed with XLH. From early childhood, she was noted to have short stature already by the age of three years, with delayed walking and poor motor endurance. Dental abscesses were frequent in childhood and adolescence. The combination of disproportionate short stature and limb deformities prompted a clinical diagnosis of pituitary dwarfism, first established in 2009 at the age of 30, when she presented to the endocrinologist for evaluation of her height and thyroid status. A decade later, in 2019, after her case was reassessed and an IGF-1 measurement was within normal limits, the diagnosis was revised to achondroplasia.

**Figure 1:**
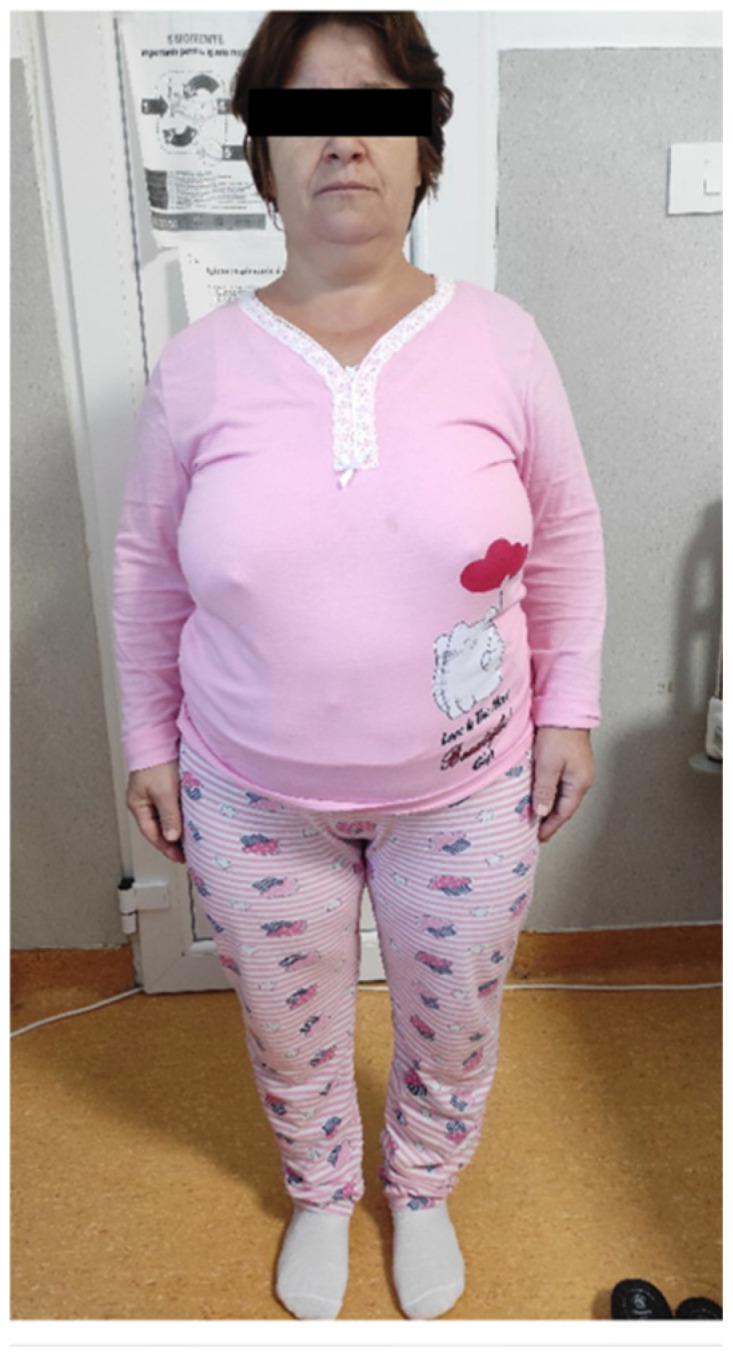
Clinical phenotype of patient S.G. (female, 43 years, index case) showing disproportionate short stature, frontal bossing and severe bilateral genu varum.

During her endocrinology assessment at the age of 43 years in 2022, she complained of long-standing diffuse bone and joint pain, predominantly nocturnal, with significant limitation of mobility. Physical examination revealed a hypersthenic constitution with a height of 139 cm (−4.35 SD), weight 75 kg, and a BMI of 37.7 kg/m^2^ (Figure 1).

#### 3.1.2. Laboratory Investigations

At initial evaluation in 2022, the patient showed persistent hypophosphatemia (serum phosphate 2.3 mg/dL; reference 2.5–5.5 mg/dL) with total serum calcium at the lower normal limit (8.1 mg/dL; 8.3–10.6 mg/dL). Alkaline phosphatase and bone-specific alkaline phosphatase were elevated at the upper reference range (121 U/L and 18.9 µg/L, respectively). Renal function was preserved, but TmP/GFR was reduced (2.03 mg/dL; 2.5–4.2 mg/dL), confirming renal phosphate wasting. After one year of conventional therapy with alfacalcidol, serum phosphate remained subnormal (2.0 mg/dL) and calcium persisted at the lower normal limit (8.2 mg/dL). Alkaline phosphatase normalized (90 U/L), and bone-specific alkaline phosphatase decreased to 12.3 µg/L. Despite this, TmP/GFR declined further to 1.76 mg/dL. At the two-year follow-up, hypophosphatemia persisted (1.7 mg/dL) and TmP/GFR decreased to 1.32 mg/dL on alfacalcidol therapy combined with intermittent oral phosphate supplementation (Table 1).

Glycosuria, aminoaciduria, and bicarbonate loss were absent, effectively excluding Fanconi syndrome.

Genetic analysis established the definitive diagnosis. Next-generation sequencing identified a heterozygous pathogenic deletion of exon 16 in the PHEX gene.

#### 3.1.3. Imaging Assessment

Imaging investigations provided further confirmation of disease burden. X-rays of the hips and knees demonstrated advanced bilateral osteoarthritis with narrowing of joint spaces, marginal osteophytes, osteosclerosis of tibial plateaus, and varus deformity of the femorotibial compartments bilaterally. No pseudofractures were observed.

DXA revealed preserved bone mineral density with lumbar and hip T-scores of +0.9 S.D., supporting the diagnosis of osteomalacia rather than osteoporosis.

Past investigations included pituitary MRI in 2019, which showed partial empty sella, but repeated hormonal studies were normal, excluding pituitary dwarfism.

#### 3.1.4. Treatment and Management

Her therapeutic history reflected decades of under recognition. As she had been considered achondroplastic, no disease-specific interventions were provided until the final diagnosis was established. In adulthood, she was managed primarily with analgesics and physiotherapy, with limited relief. Following genetic confirmation, she was referred to a multidisciplinary team including endocrinology, nephrology, rheumatology, orthopedics, and dentistry. Burosumab therapy was recommended as the first-line targeted treatment according to international guidelines, but administrative delays postponed initiation. Treatment with alfacalcidol and oral phosphate salts was started after the initial evaluation, though the patient discontinued the oral phosphate supplementation shortly afterwards, due to limited availability, minimal benefits and gastrointestinal intolerance. At her most recent evaluation, she continued alfacalcidol and remained under structured surveillance with plans for thyroidectomy for multinodular goiter and commencement of anti-FGF23 therapy thereafter. The clinical course, imaging findings, and therapeutic interventions for this case are summarized in Table 2.

### 3.2. Case Report 2—A.R., 20-Year-Old Male (Nephew of Index Patient)

#### 3.2.1. Case Presentation

A.R., a 20-year-old man, is the nephew of S.G. was first diagnosed at the age of 2 years, when parents sought medical advice for delayed walking and progressive bowing of the lower limbs. A diagnosis of familial vitamin D-resistant rickets was made, and treatment with oral phosphate and alfacalcidol (0.5 µg/day) was initiated. For approximately five years, he received multiple daily doses of phosphate along with alfacalcidol, with partial control of symptoms but frequent gastrointestinal side effects. By the age of 7, treatment was discontinued due to poor adherence and lack of specialized follow-up.

During late childhood and adolescence, growth remained impaired, with height tracking below the 3rd percentile. By adolescence, he had developed obvious lower limb deformities with pronounced genu varum and a waddling gait.

**Figure 2:**
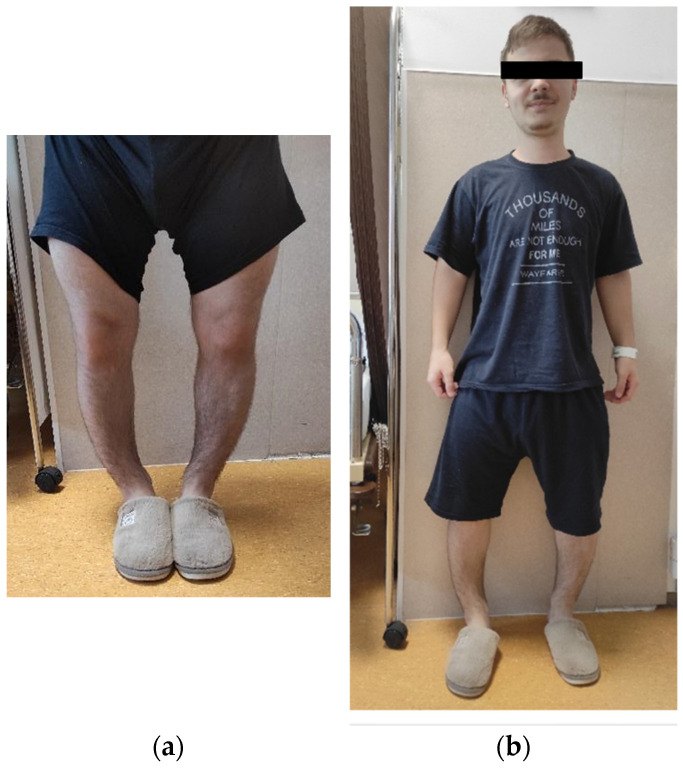
(**a**,**b**) Musculoskeletal manifestations in patient A.R. (male, 20 years, nephew of index case), including marked bowing of the femurs and tibiae with a 10.5 cm intercondylar gap and features of early bilateral coxarthrosis. Pain was elicited with passive flexion of the hips and knees.

At age 20, he presented for reassessment in light of the genetic confirmation in his aunt. His height was 154–156 cm (−2.75 SD), weight 59 kg, BMI 24 kg/m^2^, and head circumference 60.5 cm, indicating macrocephaly. Musculoskeletal examination revealed a marked genu varum with a 10 cm intercondylar gap, bowing of the femurs and tibiae, and limited range of motion of both knees (Figure 2a,b).

#### 3.2.2. Laboratory Investigations

At the initial evaluation in 2022, laboratory findings confirmed the characteristic biochemical profile of XLH. Serum phosphate was markedly reduced at 1.8 mg/dL (reference 2.5–5.5 mg/dL). Both alkaline phosphatase (ALP) and bone-specific alkaline phosphatase (BALP) were elevated. Serum creatinine and estimated GFR were normal (eGFR 130 mL/min/1.73 m^2^), but TmP/GFR was reduced (1.16 mg/dL; 2.5–4.2 mg/dL), confirming renal phosphate wasting. The patient had not received any prior therapy. During conventional treatment with oral phosphate and active vitamin D, serum phosphate increased modestly (1.8 → 2.2 mg/dL), but TmP/GFR remained low, indicating persistent renal phosphate loss. BALP decreased relative to baseline but remained above normal, reflecting ongoing high bone turnover. After one year of burosumab therapy, serum phosphate normalized (3.2 mg/dL), vitamin D metabolites improved, and ALP activity decreased, indicating partial correction of mineralization defects. However, TmP/GFR remained below reference values, suggesting incomplete restoration of renal phosphate reabsorption. At the two-year follow-up, serum phosphate remained stable within the normal range (2.5 mg/dL) and ALP was at the upper reference limit. BALP, initially elevated, decreased slightly during conventional therapy and fully normalized after two years of burosumab treatment (Table 3).

Intact FGF23 was tested at the initial evaluation and was elevated (73 pg/mL; normal 30–50 pg/mL). Endocrine screening excluded additional hormone deficiencies, with normal GH, IGF-1, cortisol, gonadotropins, prolactin, and thyroid function. These findings excluded pituitary dwarfism and Fanconi syndrome as causes of his short stature and hypophosphatemia.

Genetic analysis confirmed a hemizygous deletion of exon 16 in the *PHEX* gene, identical to the heterozygous variant found in his aunt, S.G.

#### 3.2.3. Imaging Assessment

**Figure 3:**
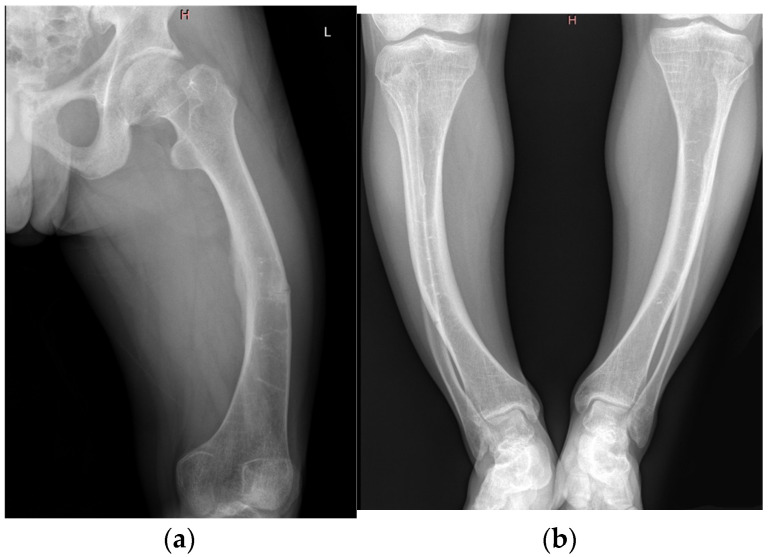
(**a**) A.R. X Rays reveals pseudofracture—middle third of the femoral shaft, pronounced bone demineralization, moderate bilateral coxarthrosis, suspicion of right sacroiliitis and (**b**) genu-varum, bilateral mild narrowing of the knee joint spaces.

X-rays of the long bones demonstrated persistent bowing of the femurs and tibiae, with cortical thickening. A pseudofracture was observed in the middle third of the femoral shaft. Radiographic suspicion of right-sided sacroiliitis was also raised. Joint imaging revealed early bilateral coxarthrosis with narrowing of the joint spaces, and knee films documented gonarthrosis (Figure 3a,b).

DXA showed lumbar spine T-score −0.8 S.D. and hip T-score −1.6 S.D., consistent with borderline osteopenia.

#### 3.2.4. Therapy

After the initial evaluation the patient was started on treatment with alfacalcidol and oral phosphate. Given his clinical, biochemical, and genetic findings, initiation of burosumab therapy was recommended. He was started on subcutaneous burosumab at 60 mg every 4 weeks, later adjusted to 70 mg/month in response to body weight and serum phosphate monitoring. Follow-up at one year demonstrated normalization of serum phosphate, improvement in TmP/GFR, and a decrease in urinary phosphate loss. Clinically, he reported gradual reduction in bone pain, improved mobility, and improved quality of life, though dental complications remained problematic. Genetic counseling was provided to inform the patient about inheritance risks and family planning considerations. Detailed chronological data on patient’s A.R. evolution are summarized in Table 4.

### 3.3. Case 3—A.D., Female, 55 Years Old

#### 3.3.1. Case Presentation

**Figure 4:**
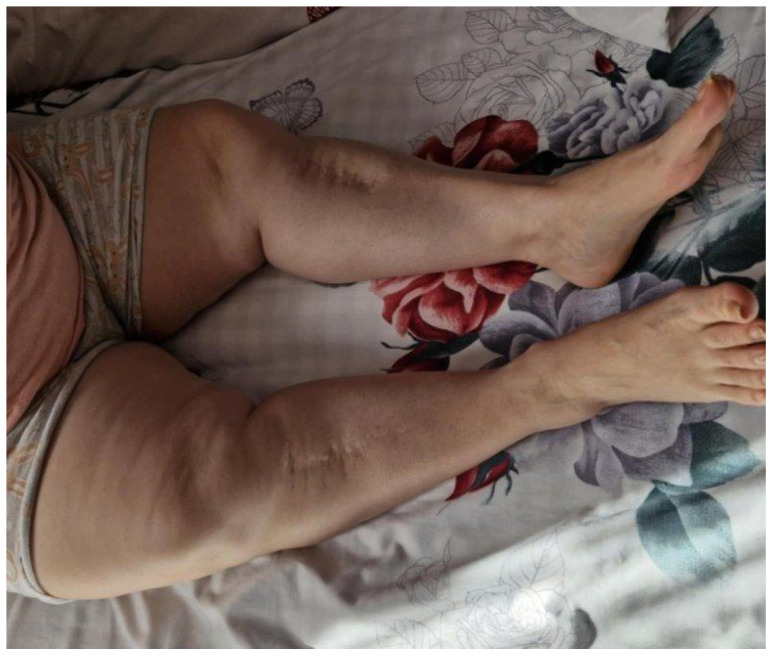
Clinical presentation of patient A.D. (female, 42 years, mother of A.R.) characterized by short stature, moderate genu varum and reduced range of motion at the knees and hips. Oral examination revealed partial edentulism and advanced periodontal disease. Comorbidities were notable. She suffered from severe bilateral coxarthrosis and gonarthrosis, confirmed radiographically. Thyroid ultrasound revealed multinodular goiter with TIRADS 4 nodules in both lobes, requiring surveillance. She also reported progressive left-sided hypoacusis, a hallmark of the disease. Neurological and psychological assessment documented mild cognitive impairment (MMSE 22/30).

A.D., a 55-year-old female, was referred to the Endocrinology Department in December 2023 after the diagnosis of XLH was established in her 20-year-old son and 43-year-old sister. She reported a lifelong history of disproportionate short stature, gait difficulties, and recurrent dental infections. On clinical assessment, the patient’s height was 131 cm (−5.73 S.D.) and her weight 71 kg, corresponding to a BMI of 41.4 kg/m^2^. She displayed disproportionate short stature, bowing of the lower limbs with an intercondylar distance of 12 cm due to genu varum, thoracic kyphosis, and wide-based gait (Figure 4).

#### 3.3.2. Laboratory Investigations

At baseline, the patient demonstrated persistent hypophosphatemia (2.8 mg/dL; reference 2.5–5.5 mg/dL) with normal calcium, elevated alkaline phosphatase (137 U/L; 38–126 U/L), and increased parathyroid hormone (97.1 pg/mL; 18.5–88 pg/mL), consistent with secondary hyperparathyroidism. Vitamin D levels were severely deficient (25-hydroxyvitamin D: 4.9 ng/mL; 30–50 ng/mL). TmP/GFR was reduced (2.41 mg/dL; 2.5–4.2 mg/dL), confirming renal phosphate wasting. Following vitamin D supplementation, 25-hydroxyvitamin D levels increased modestly (13.3 ng/mL) but hypophosphatemia worsened (2.0 mg/dL), while ALP and PTH levels remained elevated. TmP/GFR failed to normalize, indicating persistent mineralization defects. After conventional treatment with alfacalcidol, biochemical abnormalities persisted, with phosphate declining further (1.8 mg/dL) and ALP remaining elevated (139 U/L). (Table 5). Genetic testing confirmed a pathogenic *PHEX* exon 16 deletion, previously identified in her son and sister (Table 5).

#### 3.3.3. Imaging Assessment

Radiological examinations confirmed no fractures or pseudofractures, but indicated bone demineralization, bilateral coxarthrosis, gonarthrosis, marginal osteophytes and genu varum.

#### 3.3.4. Therapy

After the first evaluation, the patient received supplementation therapy to correct vitamin D deficiency, initially with cholecalciferol, then with alfacalcidol after hypophosphatemia became apparent. Treatment with oral phosphate was not administered due to limited availability. She was discharged with multidisciplinary follow-up, including endocrinology, rheumatology, orthopedics, stomatology, and otorhinolaryngology. Burosumab therapy was considered but deferred pending vitamin D repletion and further multidisciplinary evaluation. Table 6 provides a chronological overview of A.D.’s disease milestones, comorbidities, and therapeutic interventions.

## 4. Discussion

The three cases presented in this study—a 43-year-old woman (S.G.), her 20-year-old nephew (A.R.) and his mother, a 55-year-old woman (A.D.)—demonstrate the clinical heterogeneity and lifelong disease burden characteristic of XL. Although all three individuals carried the same pathogenic exon 16 deletion in the *PHEX* gene, their clinical trajectories diverged markedly, underscoring the critical importance of early diagnosis, timely therapeutic intervention, and coordinated multidisciplinary management. When left untreated, the natural course of XLH is defined by persistent renal phosphate wasting, defective skeletal mineralization, and progressive multisystemic involvement [8].

S.G. exemplifies the long-term consequences of delayed or missed diagnosis. Initially labeled as having pituitary dwarfism and later misclassified as achondroplastic, she developed disproportionate short stature, progressive skeletal deformities, recurrent dental infections and ultimately advanced bilateral coxarthrosis and gonarthrosis accompanied by disabling bone pain. These findings align with registry data indicating that untreated adults with XLH frequently experience early-onset osteoarthritis, enthesopathies, chronic pain, and significant functional impairment [1]. Importantly, she exhibited radiographic signs of osteomalacia despite normal bone mineral density on DXA, a finding that highlights the distinction between mineralization defects and osteoporosis. These findings align with histomorphometric data indicating that skeletal fragility in XLH is driven primarily by abnormal mineralization rather than reduced bone mass [9,10].

Identification of the pathogenic *PHEX* variant in S.G. not only clarified her own diagnostic uncertainty but also enabled cascade genetic testing and the timely initiation of targeted therapy in her nephew. The case of A.D. illustrates the variable penetrance and clinical expression of XLH within the same family. Although she carried the same heterozygous exon 16 deletion in the *PHEX* gene, her phenotype was comparatively milder than that of her son and her sister. She exhibited marked short stature but experienced less chronic musculoskeletal pain and fewer dental complications than her relatives. Her clinical course remained attenuated, without the profound disability observed in S.G. Surgical correction of limb deformities proved ineffective in the absence of metabolic control, emphasizing the limited value of orthopedic interventions when the underlying phosphate-wasting mechanism remains untreated.

Such intrafamilial variability has been consistently reported in large international cohorts, where phenotypic expression ranges from isolated dental abscesses to severe, rickets-like deformities [11]. Her history of conventional treatment with vitamin D supplements, followed by prolonged periods without therapy, likely contributed to the persistence of symptoms and progression of osteoarticular pathology. Similar findings have been described in adults with XLH, where conventional therapy rarely prevents long-term complications [1].

In contrast, A.R. was diagnosed early, at two years of age, with vitamin D–resistant rickets and received conventional treatment with oral phosphate and active vitamin D until the age of seven. However, poor treatment adherence and inconsistent follow-up permitted progressive complications to develop. Upon referral in early adulthood, radiographs revealed osteoarthritic changes and a femoral pseudofracture, alongside biochemical evidence of ongoing renal phosphate wasting. These findings reaffirm that conventional therapy can only partially ameliorate rickets severity and growth impairment, yet fails to prevent progressive skeletal damage. Moreover, this regimen is associated with well-recognized risks—including secondary hyperparathyroidism, nephrocalcinosis, and gastrointestinal intolerance—and does not address the underlying FGF23-mediated disease mechanism [6].

The introduction of burosumab represents a true paradigm shift in the management of XLH. A.R. commenced therapy at the age of 20 and achieved normalization of phosphate metabolism within one year, accompanied by marked symptomatic improvement and enhanced mobility. These outcomes align with findings from randomized clinical trials demonstrating superior biochemical, radiographic, and functional responses to burosumab compared with conventional therapy in both pediatric and adult XLH populations [12]. They also mirror data from international registries, which confirm improved patient-reported outcomes and quality of life under long-term burosumab treatment [5,7].

This family cluster further highlights the critical importance of genetic confirmation and cascade screening. International guidelines consistently emphasize family-based screening to facilitate early diagnosis, prompt initiation of appropriate therapy, and accurate reproductive counseling [1]. The intrafamilial variability observed—ranging from severe skeletal and dental disease in S.G. to milder phenotypes in A.D.—reflects the well-documented heterogeneity of XLH presentations, even among carriers of identical pathogenic variants [11].

Dental pathology was a major feature in all patients, consistent with literature reports that up to 80% of XLH cases present with recurrent abscesses, caries, and premature tooth loss, driven by defective dentin mineralization and enamel abnormalities [7]. Notably, dental complications may persist even after normalization of phosphate metabolism with burosumab, underscoring the need for very early therapeutic intervention to prevent irreversible dental sequelae. While current evidence demonstrates a substantial reduction in dental morbidity, it does not confirm complete resolution of these complications [13,14]. Comparable reports of familial XLH clusters underscore the wide intrafamilial variability observed in our series.

Yamamoto et al. described a three-generation Japanese family harboring a novel *PHEX* mutation with phenotypes ranging from isolated dental abscesses to severe skeletal deformities [15], findings echoed by Barros et al. in a four-generation Brazilian family carrying a single inactivating *PHEX* variant. Such variability supports the concept that modifier genes and environmental factors influence phenotypic expression despite identical genotypes [16]. Long-term natural-history studies confirm that delayed or inadequate treatment leads to accumulating complications, including early osteoarthritis, enthesopathy, chronic pain, and reduced quality of life [17]. Conventional phosphate and active-vitamin D regimens can partially improve rickets and growth but fail to prevent dental and joint deterioration while predisposing to secondary hyperparathyroidism and nephrocalcinosis [18].

Randomized phase 3 trials and subsequent real-world studies have demonstrated that burosumab normalizes phosphate metabolism, enhances mineralization, and yields superior functional and radiographic outcomes in both adults and children [19,20]. Long-term extension data further show sustained biochemical control and safety over several years [21]. Nevertheless, dental morbidity often persists despite systemic correction, as shown in recent oral-health studies reporting that structural defects of dentin and enamel may not fully resolve under burosumab [22,23]. The risks of nephrocalcinosis and secondary hyperparathyroidism, though reduced, warrant ongoing surveillance [24].

All patients also reported chronic pain, fatigue, and reduced functional capacity, findings in line with registry-based quality-of-life studies showing profound impairment in mobility, vitality and social functioning in XLH patients compared with the general population [25,26,27]. In our patients, decades of misdiagnosis compounded the clinical burden, underscoring the psychological impact of delayed recognition and the pressing need to raise awareness of XLH among pediatricians, orthopedists, and dental professionals.

Taken together, these cases reaffirm XLH as a prototypical rare disorder in which rapid advances in molecular understanding have led to the development of disease-modifying therapies that markedly transform clinical outcomes. They illustrate the striking contrast between historical results achieved with conventional therapy and the substantial improvements possible with burosumab. Continued international collaboration and prospective data collection—such as that provided by the International XLH Registry—remain essential to refining best practices and improving long-term patient outcomes [7], will be vital to refine standards of care, optimize treatment pathways and ultimately improve long-term quality of life for patients living with this rare disorder.

## 5. Conclusions

The three cases presented here illustrate the pronounced clinical heterogeneity and lifelong burden associated with XLH. Despite carrying the same pathogenic *PHEX* variant, disease trajectories diverged markedly among family members. The severe multisystem complications observed in the adult patients highlight the consequences of delayed diagnosis and the limitations of conventional therapy, whereas the stabilization and subsequent improvement achieved in the younger patient demonstrate the transformative potential of burosumab. This family cluster reinforces several key principles: the importance of maintaining a high index of suspicion for XLH across both pediatric and adult populations; the value of genetic confirmation and cascade testing within affected families; and the central role of lifelong, multidisciplinary management. Above all, timely initiation of targeted therapy remains a decisive determinant of long-term outcomes. As international registry data continue to expand, these insights will further refine best practices and improve the prognosis for patients with this rare but increasingly manageable disorder.

Learning Points

- X-linked hypophosphatemia (XLH) demonstrates marked clinical heterogeneity even within the same family, emphasizing the importance of early recognition and genetic confirmation.
- Delayed or missed diagnosis results in severe multisystem complications, including skeletal deformities, osteoarthritis, chronic pain, and complete edentulism.
- Conventional therapy with phosphate and active vitamin D provides partial benefit but fails to prevent long-term complications and carries risks such as secondary hyperparathyroidism and nephrocalcinosis.
- Burosumab directly addresses the underlying FGF23-driven pathophysiology, achieving sustained biochemical control, radiological improvement, and functional benefits in both children and adults.
- Cascade genetic screening and coordinated multidisciplinary management are essential for optimizing prognosis and guiding reproductive counseling in affected families.

## Figures and Tables

**Table 1:** Biochemical profile of patient S.G. (female, 43 years).

Test	Initial Evaluation	First Year After Treatment	Second Year After Treatment	Reference Range
Ph	2.3 ↓	2.0 ↓	1.7 ↓	2.5–5.5 mg/dL
Total Ca	8.1 ↓	8.2 ↓	8.1 ↓	8.3–10.6 mg/dL
ALP	121	90	-	38–126 U/L
BALP	18.9 ↑	12.3	-	4.5–16.9 mcg/L
PTH	35.9	42.9	42.2	18.5–88 pg/mL
25-OH Vit D	29.75 ↓	-	-	30–50 ng/mL
1,25-OH_2_D	42.8	40.2	-	19.9–79.3 pg/mL
TmP/GFR	2.03	1.76	1.32	2.5–4.2 mg/dL
eGFR	108.1	108.1	98	≥90 mL/min/1.73 m^2^
Comments	Baseline persistent hypo- phosphatemia, Vit D deficiency, ALP and marginally increased BALP	Phosphate remains low, ALP and BALP improved	Persistent hypo-phosphatemia	

Ph—Serum phosphate, Total Ca—total serum calcium, ALP—alkaline phosphatase, BALP—bone specific alkaline phosphatase, PTH—parathyroid hormone, 25-OH Vit D—25-hydroxyvitamin D (30–50 ng/mL), 1,25-OH_2_D—1,25-dihydroxyvitamin D, TmP/GFR—tubular maximum reabsorption of phosphate per GFR, eGFR—estimated glomerular filtration rate.

**Table 2:** Summary of clinical, imaging, and therapeutic findings in patient S.G.

Category	Finding	Description
General	Age/Sex	43 years, female
	Family relation	Index patient
	Genetic result	*PHEX* exon 16 deletion (heterozygous)
Clinical presentation	Onset	Early childhood—short stature, delayed walking, poor motor endurance
	Main features at clinical examination	Frontal bossing, genu varum, waddling gait, recurrent dental abscesses, chronic musculoskeletal pain; height of 139 cm (−4.35 SD), weight 75 kg, and a BMI of 37.7 kg/m^2^
	Clinical course	Misdiagnosed as pituitary dwarfism and later as achondroplasia; progressive skeletal deformities and pain in adulthood
	Comorbidities	Multinodular goiter, obesity
Imaging findings	Radiography	Bilateral coxarthrosis and gonarthrosis, tibial osteosclerosis, no pseudofractures
	DXA	Preserved bone mineral density with an osteomalacia pattern
Functional status	Mobility, dental status	Waddling gait, limited mobility, requires assistance for longer distances, complete edentulism secondary to recurrent abscesses
Treatment history	Prior therapy	None until 2022; analgesics and physiotherapy
	Conventional therapy	Alfacalcidol and oral phosphate (discontinued due to intolerance)
	Current management	Continues alfacalcidol, under multidisciplinary follow-up; planned burosumab therapy pending thyroid surgery

**Table 3:** Biochemical profile of patient A.R. (male, 20 years).

Test	Initial Evaluation	Conventional Treatment	First Year Burosumab	Second Year Burosumab	Reference Range
Ph	1.8 ↓	2.2 ↓	3.2	2.5	2.5–5.5 mg/dL
Total Ca	9.3	9.3	9.1	9	8.3–10.6 mg/dL
ALP	131 ↑	113	107	114	38–126 U/L
BALP	30.3 ↑	29.5 ↑	-	11.4	10–28.8 mcg/L
PTH	100↑	109.5 ↑	42.2	34.8	18.5–88 pg/mL
25-OH Vit D	14.5 ↓	13.3 ↓	31.7	39,4	30–50 ng/mL
1,25-OH_2_D	25.3	51.5	72.5 ↑	59.5↑	19.9–79.3 pg/mL
TmP/GFR	1.16 ↓	1.25 ↓	2.42 ↓	1.70 ↓	2.5–4.2 mg/dL
eGFR	130	135	129.9	131	≥90 mL/min/1.73 m^2^
Comments	Severe hypophosphatemia, vitamin D deficiency, high ALP/BALP, preserved renal function	Partial biochemical improvement, persistent phosphate wasting and elevated PTH	Improved phosphate and vitamin D, but TmP/GFR still low	Persistently low TmP/GFR despite stable calcium, PTH and phosphate	

Ph—Serum phosphate, Total Ca—total serum calcium, ALP—alkaline phosphatase, BALP—bone specific alkaline phosphase, PTH—parathyroid hormone, 25-OH Vit D—25-hydroxyvitamin D, 1,25-OH_2_D—1,25-dihydroxyvitamin D, TmP/GFR—tubular maximum reabsorption of phosphate per GFR, eGFR—estimated glomerular filtration rate.

**Table 4:** Summary of clinical, imaging, and therapeutic findings in patient A.R.

Category	Finding	Description
General	Age/Sex	20 years, male
	Family relation	Nephew of S.G.
	Genetic result	*PHEX* exon 16 deletion (heterozygous)
Clinical presentation	Onset	Age 2—delayed walking and progressive bowing of the lower limbs
	Main features at clinical examination	Short stature, genu varum, intercondylar gap of 10 cm, dental abscesses, height was 154–156 cm (−2.75 SD), weight 59 kg, BMI 24 kg/m^2^, and head circumference 60.5 cm
	Clinical course	Childhood diagnosis of familial vitamin D–resistant rickets; intermittent conventional therapy with poor adherence
Imaging findings	Radiography	Bowing of femurs and tibiae, pseudofracture in mid-femur
	DXA	Borderline osteopenia
Functional status	Mobility, dental status	Waddling gait; improved mobility after burosumab initiation, multiple dental abscesses requiring repeated interventions
Treatment history	Prior therapy	Oral phosphate and alfacalcidol (ages 2–7), later discontinued
	Conventional therapy	Oral phosphate and alfacalcidol (ages 2–7), later discontinued
	Current management	Burosumab since 2022 (60–70 mg monthly, adjusted to body weight)

**Table 5:** Longitudinal laboratory results in A.D. (female, 55 years).

Test	Initial Evaluation	Treatment with Cholecalciferol	Follow-Up—Treatment with Alfacalcidol	Reference Range
Ph	2.8 ↓	2.0 ↓	1.8 ↓	2.5–5.5 mg/dL
Total Ca	8.6	8.7	8.7	8.3–10.6 mg/dL
ALP	137 ↑	134 ↑	139 ↑	38–126 U/L
PTH	97.1 ↑	109 ↑	81.8	18.5–88 pg/mL
25-OH Vit D	4.98 ↓	13.27 ↓	18.13 ↓	30–50 ng/mL
TmP/GFR	2.41 ↓	1.34 ↓	1.18 ↓	2.5–4.2 mg/dL
eGFR	104	105	95	≥90 mL/min/1.73 m^2^
Comments	Hypophosphatemia with vitamin D deficiency, elevated ALP, high PTH	Partial biochemical response, persistent phosphate wasting, secondary hyperparathyroidism	Persistent hypophosphatemia ALP still elevated	

Ph—Serum phosphate, Total Ca—total serum calcium, ALP—alkaline phosphatase, PTH—parathyroid hormone, 25-OH Vit D—25-hydroxyvitamin D, 1,25-OH_2_D—1,25-dihydroxyvitamin D, TmP/GFR—tubular maximum reabsorption of phosphate per GFR, eGFR—estimated glomerular filtration rate.

**Table 6:** Summary of clinical, imaging, and therapeutic findings in patient A.D.

Category	Finding	Description
General	Age/Sex	55 years, female
	Family relation	Mother of A.R., sister of S.G.
	Genetic result	*PHEX* exon 16 deletion (heterozygous)
Clinical presentation	Onset	Early childhood with disproportionate short stature and gait abnormality
	Main features at clinical examination	Genu varum, thoracic kyphosis, recurrent dental infections, degenerative joint disease
	Clinical course	Progressive mobility limitation and pain; surgical correction of limb deformity in adulthood
Imaging findings	Radiography	Bone demineralization, bilateral coxarthrosis and gonarthrosis, genu varum
Functional status	Mobility, dental status	Reduced range of motion, moderate pain, Partial edentulism following recurrent abscesses
Treatment history	Prior therapy	None
	Conventional therapy	Cholecalciferol followed by alfacalcidol; phosphate unavailable
	Current management	Burosumab planned following vitamin D correction

## Data Availability

The patient’s data is unavailable due to privacy or ethical restrictions.
